# Lithiase biliaire et drépanocytose - à propos de deux observations à Lubumbashi (République Démocratique du Congo)

**DOI:** 10.11604/pamj.2015.21.203.6319

**Published:** 2015-07-20

**Authors:** Léon Kabamba Ngombe, Pascal Kimba Mukanya, Gray Wakamb Kanteng, Augustin Mutombo Mulangu, Oscar Luboya Numbi

**Affiliations:** 1 Université de Kamina, Faculté de Médecine, Département de Santé Publique et Médecine Interne, Unité de Toxicologie, République Démocratique du Congo; 2 Université de Lubumbashi, Faculté de Médecine, Département de Pédiatrie, République Démocratique du Congo

**Keywords:** Lithiase biliaire, douleur abdominale, échographie abdominale, enfant, drépanocytose, Lubumbashi, République Démocratique du Congo, cholelithiasis, abdominal pain, abdominal ultrasound, infant, sickle cell disease, Lubumbashi, Democratic Republic of Congo

## Abstract

Les auteurs rapportent deux cas des lithiases biliaires chez deux enfants de sexe masculin drépanocytaires, complication rare dans la littérature de notre pays. La lithiase biliaire est essentiellement consécutive à une hémolyse chronique et notamment à la drépanocytose. La douleur abdominale est le signe révélateur le plus constant de la lithiase biliaire. Le but de ce travail était de décrire cette pathologie, et de révéler les difficultés de diagnostic et de prise en charge dans un contexte comme le notre.

## Introduction

En Afrique sub-saharienne la lithiase biliaire est essentiellement consécutive à une hémolyse chronique et notamment à la drépanocytose [1, 2]. La douleur abdominale est le signe révélateur le plus constant de la lithiase biliaire [3, 4]. La lithiase biliaire est plus rare chez l'enfant que chez l'adulte, mais elle peut survenir à tout âge [5]. La fréquence de la lithiase vésiculaire de l'enfant est estimée entre 0,13% et 0,22% [6–8]. Dans notre milieu, il n'y a pas des données relatives à cette pathologie. Le but de ce travail est de décrire cette pathologie, et de révéler les difficultés de diagnostic et de prise en charge dans notre contexte.

## Patient et observation

**Figure 1:**
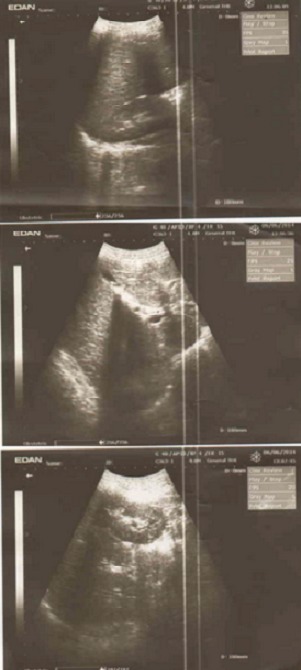
Patient A: microlithiase biliare et des microlithiases du rein droit chez un enfant drépanocytaire

Le patient A était de sexe masculin, âgé de 14 ans avec un poids de 35 Kg et une taille de 1.44 m. Il est drépanocytaire connu dès l’âge d'une année et demi, au décours d'une crise vaso-occlusive osseuse et d'une anémie. A l'admission le motif de consultation actuelle était des douleurs articulaires et abdominales atroces, ayant débuté 4 jours avant, accompagnées d'une fièvre d'allure vesperonocturne. L'examen physique révèle une sensibilité du flanc droit avec le signe de Giordano positif, une hépatomégalie à deux travers des doigts, splénomégalie type I d′hacket, et une douleur provoquée de l'hypochondre droit sans défense. Il a été transfusé une fois. Une échographie abdominale faite en urgence, montre des micros calculs rénaux du côté droit et un calcul dans la vésicule biliaire. Ainsi, le diagnostic de la lithiase biliaire et des micros calculs rénaux droit sont posés chez un enfant drépanocytaire congolais (Figure 1). Un traitement fait des antalgiques, d'anti-inflammatoires non stéroïdiens, d'antibiothérapie à large spectre (cefotaxime) et d'un antipaludéen avait été instauré car la goutte épaisse était positive.

**Figure 2:**
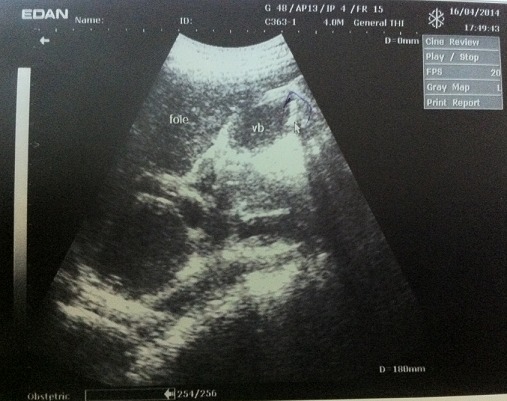
Patient B: lithiase biliare chez un drépanocytaire vue sous diferente coupe

**Figure 3:**
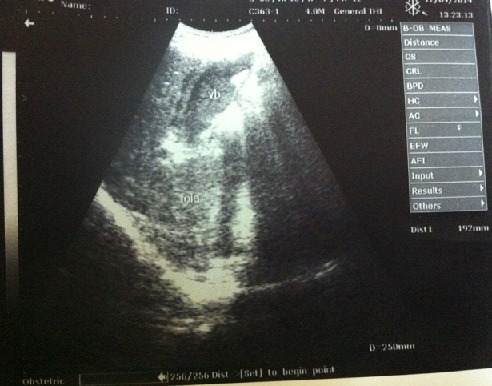
Patient B: lithiase biliare chez un drépanocytaire vue dans un autre angle

Le patient B est un adolescent de 16 ans avec un poids de 45 Kg et une taille de 1.55 m. Il est drépanocytaire connu dès l’âge de 2 ans, diagnostiqué au décours d'une crise vaso-occlusve osseuse. A l'admission le motif de consultation était des douleurs abdominales atroces, ayant débuté 7 jours avant la consultation accompagnées d'une asthénie. Il a été transfusé deux fois, 3 ans avant la consultation actuelle. L'examen physique révèle une douleur provoquée de l'hypochondre droit, un ictère franc et une hépatomégalie dépassant le rebord costal droit de 3 cm. L’échographie abdominale montre une lithiase vésiculaire et une hépatomégalie (Figure 2, Figure 3). Un traitement fait des antalgiques, d'anti-inflammatoires non stéroïdiens, d'antibiothérapie à large spectre et d'un antipaludéen avait été instauré.

Les deux patients sont suivis régulièrement dans nos services, en attente de réunir les moyens financiers pour une chirurgie et des examens anatomopathologies des calculs. Par ailleurs, il n'y a pas des modifications des résultats des échographies contrôles.

## Discussion

La drépanocytose est une maladie à caractère génétique, sa transmission se fait sur un mode autosomique dominant. De nos jours, il est connu que la lithiase biliaire est une complication fréquente chez l'enfant drépanocytaire [9–11], qui est essentiellement consécutive à une hémolyse chronique [1, 2]. La fréquence de cette lithiase biliaire est plus élevée chez les homozygotes à cause de l'importance de l'hémolyse chronique chez ces patients [12]. Nos deux cas illustrent bien cette théorie. En effet, L’âge moyen des enfants porteurs de lithiase biliaire est estimé ailleurs à 10 ans [6]. La prévalence et l'incidence augmentent avec l’âge [13]. Ces constatations sont aussi valables pour nos deux patients car aucun de ces derniers n′avait moins de 7 ans. Les données de la littérature notent une répartition de la lithiase de la vésicule biliaire chez l'enfant légèrement en faveur du sexe féminin. Il est possible que cette différence soit due à des facteurs hormonaux. En rapportant les deux cas masculins de cette étude, nous ne pouvons pas récuser la prédominance féminine rapportée par la littérature [1, 6] par insuffisance de cas de lithiase biliaire de Lubumbashi publiées localement ou ailleurs.

En ce qui concerne le mode de révélation de la lithiase biliaire, la douleur abdominale demeure le signe révélateur le plus constant de cette dernière [14, 15]. Cliniquement, nos patients ont présenté une colique hépatique; ce qui a été assez significatif pour constituer l′élément sémiologique évocateur d′une lithiase biliaire surtout qu′il s′agissait du terrain de drépanocytose connue. L′association chez le patient A d′une lithiase biliaire et des microlithiases rénales est rare dans la littérature scientifique de notre pays et attire notre attention. Par contre, dans la littérature il existe d′autres atteintes rénales associées au gène de la drépanocytose [16, 17]. Dans notre observation, nous n'avons pas eu d′arguments pouvant expliquer cette association lithiase rénale et biliaire chez notre patient A. Cependant, l′idéal serait également de pratiquer une cholécystectomie et faire l′analyse biochimique des calculs biliaires afin de connaitre le type de calculs, de même que sa nature.

Par ailleurs, certains auteurs suggèrent une échographie annuelle systématique chez les patients drépanocytaires âgés de plus de 5 ans afin de rechercher la lithiase [1, 18]. Malheureusement l′ignorance, la pauvreté et les croyances culturelles des parents d′une part et le plateau technique limitée d′autre part rendent difficile la prise en charge des lithiases biliaires chez les drépanocytaires dans notre milieu où cette maladie congénitale héréditaire représente un grand problème de santé publique.

## Conclusion

Les deux observations illustrent clairement l'intérêt et l'importance de l’échographie lors des crises douloureuses abdominales chez les patients drépanocytaires. En effet, l’échographie permet à la fois de poser le diagnostic et de suivre l’évolution. La cholécystectomie par laparoscopie reste le traitement de référence.
